# How to harvest the left internal mammary artery—a randomized controlled trial

**DOI:** 10.1093/icvts/ivae102

**Published:** 2024-05-22

**Authors:** Sofie Laugesen, Lytfi Krasniqi, Leila Louise Benhassen, Poul Erik Mortensen, Peter Appel Pallesen, Søren Bak, Bo Juel Kjelsen, Lars Peter Riber

**Affiliations:** Department of Cardio, Vascular and Thoracic Surgery, Odense University Hospital, Odense, Denmark; Department of Clinical Medicine, Faculty of Health, Odense University, Odense, Denmark; Department of Cardio, Vascular and Thoracic Surgery, Odense University Hospital, Odense, Denmark; Department of Clinical Medicine, Faculty of Health, Odense University, Odense, Denmark; Department of Clinical Medicine, Faculty of Health, Aarhus University, Aarhus, Denmark; Department of Cardiothoracic and Vascular Surgery, Aarhus University Hospital, Aarhus, Denmark; Department of Cardio, Vascular and Thoracic Surgery, Odense University Hospital, Odense, Denmark; Department of Cardio, Vascular and Thoracic Surgery, Odense University Hospital, Odense, Denmark; Department of Cardio, Vascular and Thoracic Surgery, Odense University Hospital, Odense, Denmark; Department of Cardio, Vascular and Thoracic Surgery, Odense University Hospital, Odense, Denmark; Department of Cardio, Vascular and Thoracic Surgery, Odense University Hospital, Odense, Denmark; Department of Clinical Medicine, Faculty of Health, Odense University, Odense, Denmark

**Keywords:** Coronary artery bypass, Left internal mammary artery, Graft-harvesting, flow

## Abstract

**OBJECTIVES:**

It is uncertain whether Thunderbeat has a place in harvesting the left internal mammary artery (LIMA) and whether skeletonization is superior to pedicle-harvested LIMA. Some investigations have shown improved flowrates in the skeletonized graft. The aim of this study was to compare 3 groups of harvesting techniques: Pedicled, surgical skeletonized and skeletonized with Thunderbeat in terms of flow rates in the LIMA and postoperative in-hospital outcomes.

**METHODS:**

Patients undergoing coronary artery bypass grafting with the LIMA to the anterior descending artery were randomized to pedicled (*n* = 56), surgical skeletonized (*n* = 55) and skeletonized with Thunderbeat (*n* = 54). Main outcomes were blood flow and pulsatility index in the graft.

**RESULTS:**

No statistical difference between groups regarding flow in LIMA or pulsatility index. Similarly, no difference in postoperative bleeding or days of hospitalization. The duration of harvesting was faster for the pedicled technique compared with surgical skeletonized and skeletonized with Thunderbeat [mean total min: pedicled 20.2 min standard deviation (SD) ± 5.4; surgical skeletonized 28.6 min SD ± 8.7; skeletonized with Thunderbeat 28.3 min SD ± 9.11, *P* < 0.001]. No grafts discarded due to faulty harvesting and there was no graft failure within hospital stay.

**CONCLUSIONS:**

We found no difference between the harvesting methods except for a significantly faster harvesting time with the pedicled technique. However, non-touch skeletonized LIMA harvesting with Thunderbeat seems to be an effective alternative to traditional surgical skeletonized LIMA. The future will reveal whether patency is harvesting dependent.

**Clinical trial registration:**

ClinicalTrials.gov Identifier: NCT05562908.

## Graphical abstract

**Figure:**
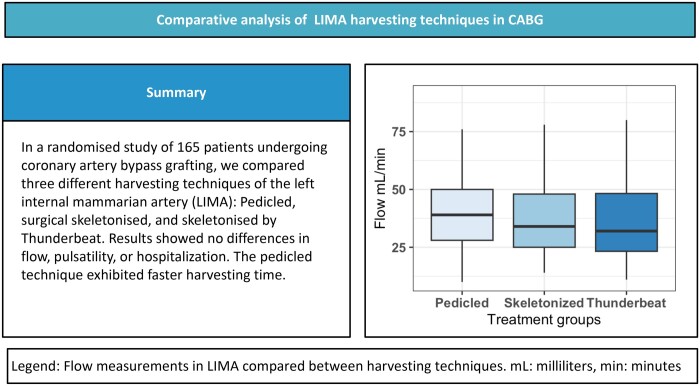

## INTRODUCTION

Coronary artery bypass grafting (CABG) has become the most prevalent procedure in heart surgery since its introduction in 1964 [1]. It remains an effective treatment for severe coronary artery disease [2].

The left internal mammary artery (LIMA) is the preferred conduit for myocardial revascularization of the front wall of the left ventricle. LIMA grafts have demonstrated clinical and survival benefits when compared with other grafts [3, 4]. They contribute to lower long-term mortality and morbidity in patients undergoing CABG, confirmed diminished rates of angina recurrence, subsequent myocardial infarction and repeat revascularization [5].

**Figure 1:**
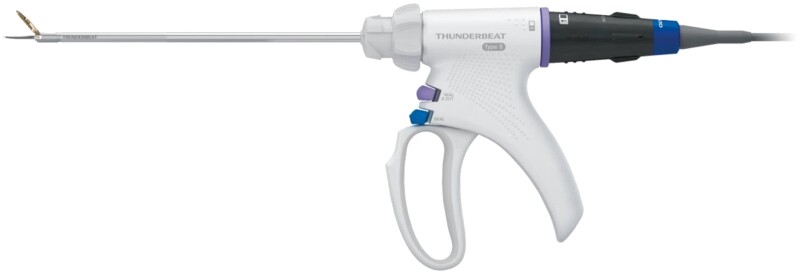
The surgical tool Thunderbeat (Olympus Medical Systems Corp., Tokyo, Japan).

There are 2 main harvesting techniques of LIMA: pedicled and skeletonized. The pedicle technique dissects the artery away from the thorax with its accompanying veins, lymphatic vessels, adipose tissue and fascia. This technique can potentially lead to significant sternal devascularization [6, 7]. The skeletonized technique requires the artery to be dissected free of all surrounding tissue. Studies have indicated that skeletonized harvesting may improve flow rates in the graft [8] and reduce chest wall dysesthesia after CABG surgery consistent with reduced intercostal nerve injury and therefore decreased risk of neuropathic chest pain [9]. Skeletonized LIMA harvesting is thought to be a more technically demanding and time-consuming technique. Concerns remain over a perceived rise in risk of injury to the LIMA graft during skeletonization, which may affect graft patency and early postoperative outcomes [10–12]. Therefore, the pedicled technique is by many surgeons the preferred harvesting method [13]. The magnitude of the potential clinical benefit from skeletonized over pedicled LIMA harvesting remains to be established [14]. New surgical energy devices are developed in the effort to decrease the need for instrument exchange when it comes to vascular ligation of side branches and hereby shorten operating time. Thunderbeat (Olympus Medical Systems Corp., Tokyo, Japan) is one of these surgical instruments (Fig. 1). It delivers simultaneously ultrasonically generated frictional heat energy and electrically generated bipolar energy. It is a multifunctional tool, and the surgeon can coagulate, cut and dissect during surgery, potentially reducing the need for tissue manipulation [15]. The instrument was developed for abdominal surgery and its abilities in LIMA harvesting have yet to be verified. To optimize the CABG procedure, an evaluation of the outcomes of the different harvesting techniques of LIMA, is called for.

We hypothesized that both the surgical skeletonized and Thunderbeat skeletonized harvesting techniques of LIMA outperform the pedicled harvesting technique in terms of flowrates and pulsatility index (PI).

Hence, the aim of this randomized controlled study was to compare 3 different harvesting techniques in regards to:

- Perioperative measurements of flow rates, PI and duration of harvesting the LIMA graft.
- The overall postoperative in-hospital outcomes such as the level of cardiac enzymes [creatine kinase-MB (CK-MB) and cardiac troponin (cTn)], postoperative bleeding and total days of hospitalization.

## PATIENTS AND METHODS

### Ethical statement

The study was approved by the Danish ethical committee—region of southern Denmark 26 October 2018. PROJECT-ID: 20180083, in accordance with the Declaration of Helsinki. Oral and written consent was obtained from all participants prior to enrolment in the study.

### Inclusion and exclusion criteria

The study was designed as a randomized controlled trial in a single centre (The Department of Cardio, Vascular and Thoracic Surgery, Odense University Hospital, Denmark) with a parallel allocation ratio of 1:1:1. Patients were viewed for admissibility to the study if they were assigned to one of the 6 project consultants and scheduled for elective on-pump CABG with LIMA to the left anterior descending artery. All 6 attending senior consultants had an operation record above 1000 cases and had a learning period of 3 Thunderbeat harvestings before entering the study. In the years prior to the study, different energy devices have been used by the consultants and both the pedicle and the surgical skeletonized harvesting techniques were routinely used by all consultants.

Patients were excluded if they were scheduled for CABG combined with other concomitant cardiac procedures (left atrial appendage occlusion was accepted); history of previous cardiac surgery, low ventricular function defined as left ventricle ejection fraction <40%, known active cancer, radiation towards the thorax, severe chronic obstructive pulmonary disease, patients unable to understand written consent, and urgent, or emergent surgery.

### Surgical procedure

#### Pedicled

**Figure 2:**
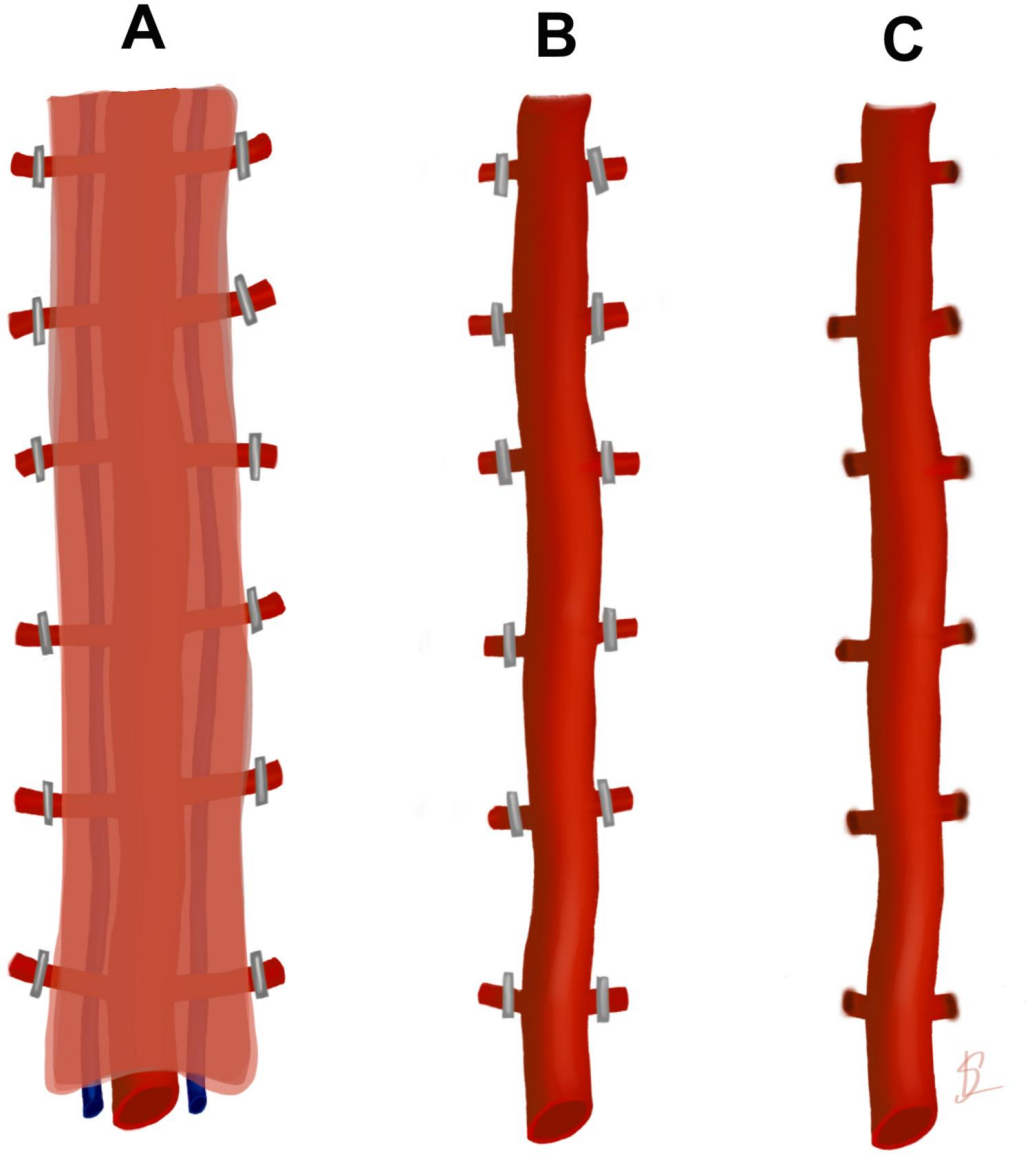
Schematic illustration of 3 different harvesting techniques for harvesting LIMA. (**A**) Pedicled, (**B**) surgical skeletonized and (**C**) skeletonized with Thunderbeat. LIMA: left internal mammary artery.

With mono-polar electrocautery a marking was made on both sides of the LIMA and its veins. The LIMA and its veins were dissected free. All side branches were closed with clips (Fig. 2).

#### Surgical skeletonized

A scissor was used to open the fascia of the LIMA. The LIMA was dissected free with scissor and forceps. Clips were then added to all side branches and divided by scissor.

#### Thunderbeat skeletonized

The fascia of LIMA was opened with Thunderbeat. The LIMA was dissected free with Thunderbeat including ligation of all side branches.

In all 3 harvesting techniques: The LIMA was divided distally by adding clips on the peripheral part of the vessel and proximately dividing by scissor once the full length of the LIMA was achieved. A vessel clamp was positioned distally, and the skeletonized LIMA was placed with a cloth containing papaverine in the jugular cavity.

### Outcome measures

The duration of LIMA harvesting was defined as time from insertion to removal of the LIMA-harvesting sternal retractor. The graft flow and pulsatility index (PI) were obtained with transit time flowmetry (Sono TT flowlab). The measurements were done with probe size 3 or 4 and after weaning off the extracorporeal circulation with a systolic pressure targeted at 100 mmHg.

Postoperative bleeding was measured from the end of the operation to removal of the mediastinal drains in the intensive care unit. CK-MB (µg/l) and cTn (ng/l) were assessed 4 h post-removal of the aortic cross-clamp. Total days of hospitalization was calculated as days from the operation date and the last day included the patient’ discharge day from hospital.

The primary outcome for sample size calculation was LIMA flow. Quality data from our institution showed that the mean intraoperative transit time flow of the LIMA graft was 35 ml/min.

We obtained data from our national database, to see if there were any differences in flow registered, indicating a 10% increased flow in skeletonized grafts. To detect an absolute difference of 4 ml/minute between skeletonized and pedicled LIMA, with the assumption of a mean flow of 35 ml/minute in the pedicled group, the mean variance of 8 and at an alpha level of 0.05 and 80% power. This gave an estimated sample size by one-way analysis of variance of 123 patients with 41 patients in each of the 3 groups required.

### Randomization

Patients were randomized 1 week prior to operation by the digital data-collection solution REDcap. Stratified regarding beta-blockers and diabetic mellitus to one of the 3 groups. The project leader created the data-collection platform in REDcap and was responsible for assigning patients to the project. After randomization, the attending consultant informed the patient of the harvesting method. Data collector and outcome adjudicator were blinded.

### Statistical analysis

Baseline data was obtained from patient records (Table 1). A scrub nurse registered perioperative data such as graft flow and pulsatility index (PI) during the operations. Continues variables were presented as means with standard deviation (SD). Categorical variables were described as percentages. To determine any statistically significant differences between the means of the 3 groups a one-way analysis of variance was applied (Table 2). Results were further analysed with multivariable linear regression models where the pedicled technique was established as reference group. Variables were decided upon prior to data analysis and included based on clinical reasoning. All analyses adjusted for the impact of the surgeons performing the operations. An overview of variables is provided in Table 3. The influence of outliers was evaluated but not excluded from the results. Diagnostic control analyses were made of all the model assumptions. Robust standard errors were calculated along with associated values of confidence intervals and *P*-values when the assumption of homoscedasticity was violated (Table 3). A *P*-value of below 0.05 was considered statistically significant. All statistical analyses were performed in R 4.1.2 and RStudie 20.02.3, PBC.

**Table 1:** Demographic and clinical patient characteristics by treatment group

Characteristic	Pedicled (*n* = 56)			Surgical skeletonized (*n* = 55)			Skeletonized Thunderbeat (*n* = 54)		
	*n*	Mean/%	SD	*n*	Mean/%	SD	*n*	Mean/%	SD
Age		66.6	8.7		66.7	9.01		64.7s	9.4
Men (%)		82%			91%			93%	
BMI		28.4	4.6		28.9	4.0		28.4	4.2
BSA (m2)		2.1	0.3		2.1	0.2		2.1	0.2
EF		56.1	5.9		56.0	6.12		55.8	7.2
Smoking (%)									
Active	9	17%		13	24%		10	19%	
Former	31	56%		26	48%		23	43%	
Never	15	28%		16	29%		21	39%	
Creatinine (µmol/l)		87.4	23.1		89.6	25.7		86.2	17.3
eGFR (ml/min/1.73 m2)		76.7	15.5		76.0	16.4		78.1	12.2
Prior PCI (%)	9	16%		8	15%		6	11%	
Prior stroke (%)	6	11%		10	18%		5	9%	
Anticoagulants (%)									
Warfarin	0			0			0		
NOAC	2	4%		0			2	4%	
Antiplatelet	12	21%		15	27%		14	26%	
None	42	75%		40	73%		38	70%	
Lipid-lowering agent (%)	47	84%		49	89%		44	82%	
NYHA class (%)									
NYHA 1	38	67%		32	58%		31	57%	
NYHA 2	16	29%		18	33%		19	35%	
NYHA 3	2	4%		5	9%		4	7%	
NYHA 4	0			0			0		
CCS class (%)									
CCS 1	6	11%		5	9%		7	13%	
CCS 2	29	52%		35	64%		33	61%	
CCS 3	20	36%		11	20%		9	17%	
CCS 4	1	2%		4	7%		5	9%	
Euroscore II		1.18	0.87		1.30	0.99		1.07	0.62
Stratification^a^ (%)									
Beta-blockers	23	41%		22	40%		22	41%	
Diabetes	4	7%		5	9%		5	9%	
Both	4	7%		3	6%		3	6%	
None	25	45%		25	46%		24	44%	
Grafts in total^b^ (%)									
1 graft	1	2%		2	4%		1	2%	
2 grafts	11	20%		11	20%		12	22%	
3 grafts	30	54%		27	49%		28	52%	
4 grafts	13	23%		12	22%		12	22%	
5 grafts	1	2%		3	6%		1	2%	

All continues variables were expressed as mean ± SD and categorical variables were described as percentages.

a Randomization was stratified according to patient status of diabetes, use of beta-blockers, both or none.

b The total number of graft that patients received during CABG.

BMI: body mass index; BSA: body surface area measured in m^2^; CABG: coronary artery bypass graft; EF: ejection fraction; eGFR: Estimated glomerular filtration rate, PCI: percutaneous coronary intervention; NOAC: Non-vitamin K antagonist oral anticoagulants; NYHA: New York Heart Association Classification; CCS: Canadian Cadiovascular Society Angina Grade; SD: standard deviation.

**Table 2:** Peri- and postoperative outcomes between the 3 different harvesting techniques

Outcome variables	Pedicled (*n* = 56)		Surgical skeletonized (*n* = 55)		Skeletonized Thunderbeat (*n* = 54)		*P*-value
	Mean	SD	Mean	SD	Mean	SD	
Flow in LIMA (ml/min)	39.1	16.0	37.1	15.1	36.9	19.5	0.76
PI in LIMA	1.9	0.5	2.1	0.9	2.0	0.8	0.21
Time to harvest LIMA (min)	20.17	5.4	28.6	8.7	28.3	9.1	<0.001
Cross-clamp time (min)	46.6	17.4	48.6	16.3	44.1	13.5	0.36
ECC time (min)	81.4	20.8	82.6	25.2	78.3	17.6	0.56
Postoperative bleeding (ml)	534.3	301.6	678.3	560.7	599.3	428.0	0.23
CK-MB levels (µg/l)	44.5	35.4	41.4	30.6	43.2	21.1	0.86
cTn levels (ng/l)	1506.0	951.3	1392.7	843.4	1513.2	763.7	0.71
Intensive care unit (days)	1.3	1.1	1.2	0.7	1.1	0.6	0.46
In hospital stay (days)	6.4	4.4	5.9	1.9	5.9	2.9	0.57

All variables were continuous variables and were expressed as mean ± SD. Equality of means was analysed with one-way ANOVA.

ANOVA: analysis of variance; CK-MB: creatine kinase-MB; cTn: Cardiac troponin T; LIMA: left internal mammary artery; PI: pulsatility index; ECC: extracorporeal circulation; SD: standard deviation.

**Table 3:** Clinical outcomes and results

	Adjusted coefficients	Adjusted 95% CI	Adjusted *P*-value
Flow in LIMA^a^ (ml/min)			
Pedicled	Reference		
Surgical skeletonized	1.4	−6.7 to 9.5	0.72
Skeletonized Thunderbeat	2.7	−5.3 to 10.7	0.50
PI in LIMA^a^			
Pedicled	Reference		
Surgical skeletonized	0.2	−0.2 to 0.5	0.34
Skeletonized Thunderbeat	0.2	−0.2 to 0.5	0.36
Time to harvest LIMA^b^ (min)			
Pedicled	Reference		
Surgical skeletonized	8.1	4.4 to 11.8	<0.001
Skeletonized Thunderbeat	7.9	4.3 to 11.6	<0.001
Cross-clamp time^c^ (min)			
Pedicled	Reference		
Surgical skeletonized	−0.6	−5.2 to 4.1	0.81
Skeletonized Thunderbeat	−2.6	−7.1 to 2.0	0.26
ECC time^c^ (min)			
Pedicled	Reference		
Surgical skeletonized	−1.6	−8.2 to 4.9	0.63
Skeletonized Thunderbeat	−3.1	−9.6 to 3.3	0.34
Postoperative bleeding^d^ (ml)			
Pedicled	Reference		
Surgical skeletonized	75.6	−72.6 to 223.8	0.31
Skeletonized Thunderbeat	54.1	−93.1 to 201.4	0.46
CK-MB levels^e^ (µg/l)			
Pedicled	Reference		
Surgical skeletonized	−1.0	−13.7 to 11.6	0.87
Skeletonized Thunderbeat	−0.4	−10.5 to 9.6	0.93
cTn levels^f^ (ng/l)			
Pedicled	Reference		
Surgical skeletonized	−31. 2	−359.3 to 297.0	0.85
Skeletonized Thunderbeat	40.5	−260.6 to 341.5	0.79
Intensive care unit^g^ (days)			
Pedicled	Reference		
Surgical skeletonized	−0.1	−0.5 to 0.2	0.24
Skeletonized Thunderbeat	−0.2	−0.5 to 0.1	0.35
In hospital stay^f^ (days)			
Pedicled	Reference		
Surgical skeletonized	−0.5	−1.7 to 0.7	0.40
Skeletonized Thunderbeat	−0.6	−2.0 to 0.8	0.38

The pedicled technique was set as reference group also referred to as the intercept (a mathematical constant with no clinical interpretation). The adjusted coefficients are the difference from the pedicled technique in the linear regression model after adjustments for variables. SE is the estimated precision of the adjusted coefficients; 95% CI is the 95% CIs for the adjusted coefficients.

a Adjusted for surgeon, BMI and systolic blood pressure.

b Adjusted for surgeon.

c Adjusted for surgeon and number of grafts.

d Adjusted for surgeon, BMI and ECC time.

e Adjusted for surgeon and cross-clamp time. The presented results are based on robust SE.

f Adjusted for surgeon and cross-clamp time. The presented results are based on robust SE.

g Adjusted for surgeon and Euroscore II. The presented results are based on robust SE.

h Adjusted for surgeon and Euroscore II. The presented results are based on robust SE.

BMI: body mass index; CI: confidence interval; CK-MB: creatine kinase-MB; cTn: cardiac troponin T; ECC: extracorporeal circulation; LIMA: left internal mammary artery; PI: pulsatility index; SD: standard deviation; SE: standard error.

## RESULTS

### Patients’ characteristics

**Figure 3:**
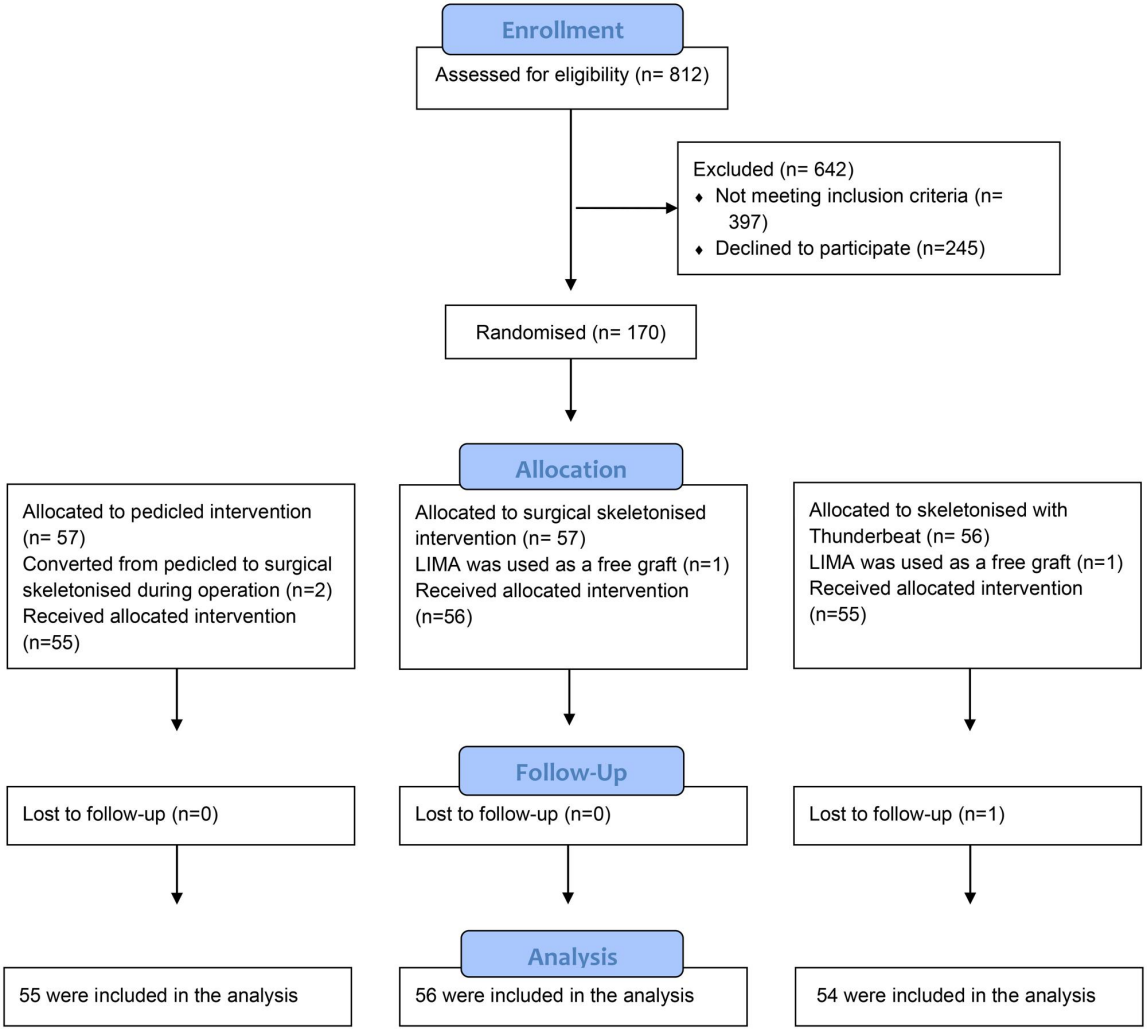
Flow diagram for the inclusion of patients.

A total of 812 patients were reviewed for admissibility (Fig. 3). 170 consecutive patients agreed to participate in the study. Two patients were excluded from the study perioperative because LIMA was used as a free graft due to anatomically changes presumably caused by previous trauma. To obtain length, the harvesting method changed from pedicled to surgical skeletonized during the operation in 2 cases, and 1 patient regretted participation in the study (Fig. 3). A total of 165 patients were included in the analysis. The average age was 66 years (95% confidence interval: 64.8–67.3) and 77% of patients were men. Operations took place between April 2019 to April 2021. Randomization was performed with 55 patients receiving pedicled LIMA, 56 patients receiving surgical skeletonized LIMA and the remaining 54 patients received LIMA skeletonized with Thunderbeat. All patients received a single LIMA to left anterior descending artery. Baseline characteristics are shown in Table 1.

### Results of measured outcomes

There was no statistical difference between groups regarding LIMA flow and PI before or after adjustment for variables (Tables 2 and 3). Both surgical skeletonized and Thunderbeat skeletonized had increased postoperative bleeding (mL) compared to the pedicle technique, although not statistically significant. Cross-clamp time and ECC time for both surgical skeletonized and Thunderbeat skeletonized were faster than the pedicled technique, but not statistically significant. There was no significant difference in Ck-MB or cTn between the 3 groups. Likewise, the length of stay at the intensive care unit did not differ between the groups (Tables 2 and 3). The time for harvesting LIMA was 8 min faster in the pedicle group when compared with surgical skeletonized and Thunderbeat skeletonized (mean total min: pedicled 20.2 min SD ± 5.4; surgical skeletonized 28.6 min SD ± 8.7; skeletonized with Thunderbeat 28.3 min SD ± 9.11, *P* < 0.001) (Tables 2 and 3).

No LIMA grafts were discarded due to faulty harvesting. Seven patients were re-operated due to bleeding, but none because of the harvesting technique of LIMA (Table 4). Three patients were re-operated due to malfunction of the vein graft, which was verified by coronary angiogram, but none because of LIMA failure. A total of 16 patients had clinical symptoms of pleural effusion on the left side, why pleurocentesis was performed. No statistically significant difference in the volume of the plaural exudate was found between groups (Table 4) No instances of sternal wound infection were detected.

**Table 4:** Postoperative characteristics between treatment groups

Characteristic	Pedicled (*n* = 56)			Surgical skeletonized (*n* = 55)			Skeletonized Thunderbeat (*n* = 54)		
	*N*	Mean/%	SD	*N*	Mean/%	SD	*N*	Mean/%	SD
Re-operation due to bleeding (%)	0			5	9%		2	4%	
Re-operation due to ischaemia (%)	2	4%		1	2%		0		
Pleurocentesis (%)	4	7%		5	9%		7	13%	
Pleura fluid (ml)		1162.5	213.6		1040.0	391.2		785.7	343.7

Continuos variables were presented as means ± SD. Categorical variables were described as percentages.

SD: standard deviation.

## DISCUSSION

For years, LIMA has been the recommended standard for grafting the left descending artery. However, the best technical way of harvesting LIMA is still not clear. In this randomized controlled trial, we found no significant difference between pedicled, surgical skeletonized and Thunderbeat skeletonized harvesting methods regarding flow in LIMA, PI, postoperative bleeding, CK-MB, cTn, postoperative stay in intensive care unit and total days of hospitalization.

Transit time flowmetry measurement (TTFM) is often used to evaluate graft flow during the operation and can help detect early graft failure[16, 17]. Studies report that TTFM is associated with factors including body mass index, systolic blood pressure and cardiac index [18]. We adjusted for these factors in the multiple regression analysis without finding any statistically significant difference between the 3 groups.

Several studies have highlighted the advantage of skeletonized LIMA primarily because of better flow in skeletonized LIMA compared to pedicled LIMA [19, 20], but conflicting studies exist [21]. Sá *et al.* [8] observed a difference between results depending on study design; non-randomized studies demonstrated a superiority regarding skeletonized LIMA while randomized studies did not. Those results match our findings.

Other studies that investigated the difference between pedicled and skeletonized harvesting techniques found that skeletonized LIMA resulted in lesser postoperative bleeding [22]. In theory the different harvesting techniques could have a direct impact on blood loss due to ligation of the side branches of vessels. Another possibility is that the harvesting techniques may have different impact on the coagulation system affecting postoperative bleeding. Though, this was not something we were able to demonstrate in our study. The variances between groups were not large enough to have clinical significance.

We measured myocardial injury markers such as CK-MB and cTn and found no difference between the 3 groups. Mazur *et al.* [22] tested the pedicled harvesting technique against the skeletonized technique and found a higher increase in CK-MB over the first 12 h after surgery for the pedicled group. These differences were mostly statistically insignificant. Previous studies have shown that CK-MB needs to be elevated 10 times the upper reference limit [23] and cTn 57 times the upper limit [24] to have an impact on postoperative outcomes after CABG, so these small differences between groups have no clinical relevance.

A total of 7 patients were re-operated due to postoperative bleeding; 2 were operated with Thunderbeat and 5 were operated with surgical skeletonized technique. Even though the skeletonized technique was overrepresented, there are too few events to draw any definite conclusions.

Our study showed a clear time advantage when harvesting LIMA with a pedicled technique. Both surgical skeletonized and skeletonized with Thunderbeat were significantly slower than the pedicled technique (Table 3). It did not, however, have an impact on the overall ECC time in the 3 groups. In our findings harvesting LIMA skeletonized with Thunderbeat performed equally as good as the surgical skeletonized LIMA harvesting, hereby demonstrating, that harvesting LIMA with Thunderbeat is an efficient alternative to harvesting LIMA in a traditional surgical skeletonized way. We did not demonstrate an advantage of using the Thunderbeat technique compared with normal surgical technique for CABG procedures.

### Limitations

In this study, there are fewer patients included than in some of the larger observational multicentre studies. As a single centre study the execution of LIMA harvesting is held in few and experienced hands. All surgeons had opportunity to view and comment the surgical protocol prior to the initiation of the study. They all accepted the surgical protocol without regard to personal preferences when harvesting LIMA, which guaranteed a uniform harvesting technique. Mean flow of the graft was employed to calculate the sample size. This may be adequate for this end-point, but the power of the final sample for other secondary outcomes is low, and the study might be underpowered for making inference for the secondary end points.

The most accurate method to evaluate the functionality of the graft would be with coronary angiogram. This was unfortunately not technically possible in this study. Instead TTFM was applied to evaluate the functionality of the LIMA graft. Several studies have reported that TTFM can foresee graft failure[16, 17] and A recent review of the TTFM measurement concluded that TTFM improved CABG outcome[25]. TTFM is easily accessible, and it is measured at our institution on a standard basic to assess the graft functionality.

A noteworthy proportion of patients rejected participation in the study, with only 170 out of 415 patients (41%) eligible for inclusion. This could cause inclusion bias, but no difference was found in baseline in-between the 3 arms and non-included patients. The inclusion period was also during the Covid pandemic, which could influence the inclusion of patients. The study was unblinded as the patients’ assigned methods of conduit harvesting technique was naturally known to the surgeons, care providers and patients. Therefore, bias may be present in the postoperative treatment of the patients. Parameters such as conduit length can also affect flow parameters, but we adapted the length of the LIMA graft to each individual patient and had no problems with the length regardless of which group the patient belonged to, although you can generally harvest longer grafts with the skeletonized harvesting technique [10].

In most cases, the pleural space was opened during the harvesting of LIMA, why pleura transudate may have contributed to the overall drainage amount measured in the hours after surgery.

The outcomes of this study were based on data collected in the perioperative and postoperative period during the patients’ hospital stay. We cannot make inference about any potential benefits from different harvesting techniques on graft patency based on the current data, but a follow-up study will be performed on our study population.

## CONCLUSION

In this randomized, controlled study of 165 consecutive CABG patients, we found no difference in-between the harvesting methods regarding flow, pulsatility index, postoperative bleeding, total days of hospitalization or adverse events. In accordance with our findings, these 3 methods seem to be equal in performance. Only duration of LIMA harvesting was lower in the pedicled group compared with the 2 skeletonized groups. The future will show if patency is harvesting-dependent.

## Data Availability

The data that support the findings of this study are available from the corresponding author, upon reasonable request.

## Notes

Presented at the 36th EACTS Annular Meeting 5-8 October 2022 in Milan, Italy.

## ABBREVIATIONS

CABG: Coronary artery bypass grafting
CK-MB: Creatine kinase-MB
cTn: Cardiac troponin T
LIMA: Left internal mammary artery
SD: Standard deviation
TTFM: Transit time flowmetry measurement
